# Supplementary material for: Contingent convertible bonds in financial networks
Source: Sci Rep. 2023 Dec 15;13:22337. doi: 10.1038/s41598-023-48228-9 (PMC10724134; doi:10.1038/s41598-023-48228-9)
Supplement: Supplementary file 1 — Supplementary Information. [file 41598_2023_48228_MOESM1_ESM.pdf]

Supplementary Material: Contingent Convertible Bonds in Financial Networks

Giovanni Calice[1], Carlo Sala[2], and Daniele Tantari[3,*]

[1]Bangor Business School, Bangor University, Bangor, LL57 2DG, United Kingdom
[2]Department of Economics, Finance and Accounting, ESADE Business School (Ramon Llull University), Avenida de Torreblanca 59, Barcelona, Spain
[3]Mathematics Department, University of Bologna, Piazza di Porta S. Donato 5, 40126, Bologna, Italy.
[*]daniele.tantari@unibo.it

ABSTRACT

This Supplementary Material provides all the proofs of the main Theorems in the main text.

1 Proof of Theorem 1

1.1 Ring Network

In this case $y_{ij} = y(\delta_{j,i+1} + \delta_{j,i-1})$. We can study analytically the equilibrium of the system after an idiosyncratic shock of size ε on one bank: $z = (a-\varepsilon, a, \dots, a)$, $a > s$ and $\varepsilon > (a-s)$. Denoting $\phi^\star$ the equilibrium we have that $\phi_1^\star \leqslant \phi_2^\star \leqslant \dots \leqslant \phi_n^\star$. Let

$$\bar{n}(\phi^\star) = \max\{i : \phi_i^\star < 1\} \tag{1}$$

the number of insolvent banks. As soon as $\bar{n} < n$, it should be $\phi_n = 1$ and thus:

$$\begin{aligned}
\phi_1 &= f_{y,s}(y + a - \varepsilon) \\
\phi_i &= \phi_{i-1} + \frac{a-s}{y} = \phi_1 + (i-1)\frac{a-s}{y}, \quad i = 2, \dots, \bar{n} \\
\phi_i &= 1, \quad i > \bar{n}.
\end{aligned}$$

It holds as soon as $\bar{n} < n$, i.e. $f_{y,s}(y + a - \varepsilon) + (n-1)\frac{a-s}{y} > 1$, whose solution is

$$\varepsilon < \varepsilon^\star = n(a-s) \qquad \text{or} \qquad y < y^\star = (n-1)(a-s), \tag{2}$$

exactly as in[1]. This is the equilibrium in the small shock ($\varepsilon < \varepsilon^\star$) or low exposure ($y < y^\star$) regime, where $\varepsilon^\star$ is the total system' liquidity in absence of shocks. In this regime the extension of the contagion is, from Equation 2,

$$E(\phi^\star) = \bar{n}(\phi^\star)/n = \lfloor 1 + \frac{y}{a-s}(1 - f_{y,s}(y + a - \varepsilon))\rfloor/n, \tag{3}$$

which is linearly step-wise increasing with ε until $\min(\varepsilon^\star, y + (a-s))$. Analogously the total distress is

$$D(\phi) = \frac{\|1-\phi^\star\|}{n} \approx \frac{\bar{n}(\phi)(1-\phi_1^\star)}{2n} \tag{4}$$

which grows quadratically in ε up to the transition. On the contrary, when $\varepsilon > \varepsilon^\star$ and $y > y^\star$, it is $\bar{n} = n$. In this regime it should be at the same time $\phi_n = \phi_1 + (n-1)\frac{a-s}{y}$ and $\phi_1 = (\phi_n + \frac{a-s-\varepsilon}{y})^+$. The only possibility is that:

$$\begin{aligned} \phi_1 &= 0 \\ \phi_i &= (i-1)\frac{a-s}{y}, \quad i > 1. \end{aligned}$$

This solution is independent from ε since the distress propagation is already maximal: $E(\phi^\star) = 1$ and $D(\phi^\star) = 1 - \frac{(a-s)(n-1)}{2y} = 1 - \frac{1}{2}\frac{y^\star}{y}$.

1.2 Fully connected network

In this case, every pair of banks is connected, and the junior liability of a bank is equally distributed among its neighbors, i.e. $y_{ij} = \frac{y}{n-1}, \forall i \neq j$. Again, we can study analytically the equilibrium of the system after an idiosyncratic shock of size ε on one bank: $z = (a-\varepsilon, a, \ldots, a)$, $a > s$ and $\varepsilon > (a-s)$. In fact, in this case, all the updating rules in Eq. (3) of the Main text for non shocked banks have the same structure. Thus, we can make an ansatz for the equilibrium and search for a solution of the form:

$$\phi_i = \begin{cases} \phi_s & \text{if } i = 1, (\text{ shocked bank}) \\ \phi_{ns} & \text{if } i > 1, (\text{ non shocked bank}), \end{cases} \tag{5}$$

where (ϕ_s, ϕ_{ns}) satisfying:

$$\begin{aligned} \phi_s &= f_{ys}(a - \varepsilon + y\phi_{ns}) \\ \phi_{ns} &= f_{ys}(a + \frac{y}{n-1}\phi_s + y\frac{n-2}{n-1}\phi_{ns}). \end{aligned} \tag{6}$$

Equation (6) do not admit solutions if both $\phi_s, \phi_{ns} \in (0,1)$. The two possibilities are

$$\begin{aligned}
\phi_{ns} = 1 \quad &\Longrightarrow \quad \phi_s = \left(1 - \frac{\varepsilon - (a-s)}{y}\right)^+ \\
\phi_s = 0 \quad &\Longrightarrow \quad \phi_{ns} = (n-1)\frac{(a-s)}{y} = \frac{y^\star}{y}.
\end{aligned} \tag{7}$$

The latter is admissible as soon as $y > y^\star$ and $\phi_s = 0$, implying $\varepsilon > n(a-s) = \varepsilon^\star$, thus corresponding to the large shock large exposure regime. On the contrary, $y < y^\star$ or $\varepsilon < \varepsilon^\star$, the first solution holds, corresponding to an equilibrium where the shocked bank is insolvent while the rest of the system has absorbed the distress. In this second case, the solution is independent from ε for $\varepsilon > y + (a-s)$, corresponding to the maximum distress that bank 1 can propagate given its (limited) debt. The extension of contagion and overall financial distress can be easily computed starting from the analytical solutions 7. In particular, their maximum values in the large shock regime are $E(\phi^\star) = 1$ and $D(\phi^\star) = 1 - (\frac{1+(n-1)y^\star/y}{n}) \approx 1 - y^\star/y < D^{ring}$.

2 Existence of a unique equilibrium

In this section we prove that in the presence of Coco's the system admits an unique equilibrium in terms of the fitness vector. Given $\tau \in [0,1)$, $\eta \in [0,1]$, $e \in \mathbb{R}^n$ and S a $n \times n$ stochastic matrix, i.e. $S_{ij} \in [0,1]$ and $\sum_i S_{ij} = 1$ $\forall j = 1,\dots,n$, let's define the continuous map $F : [0,1]^n \longrightarrow [0,1]^n$ as

$$\phi \longrightarrow F(\phi) = \eta + (1-\eta)\min[1, e + (1-\tau)S\phi]^+. \tag{8}$$

If we identify $S_{ij} = y_{ij}/y$ and $e_i = [(1-\tau)z - s]/y$, this is exactly the general propagation rule in the presence of Cocos and equity liquidation. We have the following

Theorem 2.1. *F admits an unique fixed point in* $[0,1]^n$.

Proof. First of all F is a continuous map on a convex compact set, therefore a fixed point exists for the Brouwer theorem. The uniqueness comes from the fact that F is a contractive map. In fact, given $\phi, \hat{\phi} \in [0,1]^n$ it holds

$$\begin{aligned}
\|F(\phi) - F(\hat{\phi})\|_1 \quad &= \quad (1-\eta)\left\|\min[1, e + (1-\tau)S\phi]^+ - \min[1, e + (1-\tau)S\hat{\phi}]^+\right\|_1 \\
&\leqslant \quad (1-\eta)\left\|\min[1, e + (1-\tau)S\phi] - \min[1, e + (1-\tau)S\hat{\phi}]\right\|_1 \\
&\leqslant \quad (1-\eta)(1-\tau)\left\|S(\phi - \hat{\phi})\right\|_1,
\end{aligned} \tag{9}$$

where we introduced $\|x\|_1 = \sum_i |x_i|$ and where we used that $|v^+ - w^+| \leqslant |v-w|$ and $|\min(u,v) - \min(u,w)| \leqslant |v-w|$. Since $\left\|S(\phi - \hat{\phi})\right\|_1 \leqslant \|S\|_1 \|\phi - \hat{\phi}\|_1$ and $\|S\|_1 = \max_j(\sum_i |S_{ij}|) = 1$, it holds $\forall \phi, \hat{\phi} \in [0,1]^n$

$$\|F(\phi) - F(\hat{\phi})\|_1 \leqslant (1-\eta)(1-\tau)\|\phi - \hat{\phi}\|_1 < \|\phi - \hat{\phi}\|_1, \tag{10}$$

if $\tau \in (0,1]$. The case $\tau = 0$ (and $\eta = 0$) corresponds to the propagation rule in absence of Cocos that has been proved to have a unique equilibrium under mild conditions in[1,2]. □

3 Proof of Theorem 2

First, we need the following propositions:

Proposition 3.1. *In a ring network with $z = (a-\varepsilon, a, \ldots, a)$ it holds $\forall i = 1, \ldots, n-1$*

$$\phi_i^\star = 1 \implies \phi_{i+1}^\star = 1.$$

Proof. For any $i = 1, \ldots, n-1$, $\phi_i^\star = 1$ means that $((1-\tau)a - s)/y + (1-\tau)\phi_{i-1}^\star - \delta_{i1}(1-\tau)\varepsilon/y \geqslant 1$, i.e. $((1-\tau)a - s)/y \geqslant 1 - (1-\tau)\phi_{i-1}^\star \geqslant 1 - (1-\tau)$.]= Thus:

$$(1-\tau)\phi_i^\star + \frac{(1-\tau)a - s}{y} = (1-\tau) + \frac{(1-\tau)a - s}{y} \geqslant 1,$$

implying $\phi_{i+1}^\star = 1$. At $\tau = 0$ the proposition is true simply because the sequence $\phi_i^\star$ is weakly increasing. □

Proposition 3.2. *For any $b \in \mathbb{R}$, $\varepsilon \geqslant 0$ and $\lambda \in [0,1]$, given $\phi(y,\varepsilon) = (1-\tau)(1-\varepsilon/y) + b/y$, then the portion of the positive (y,ε) plane defined by the stability condition:*

$$(1-\lambda)\min(\phi(y,\varepsilon),1)^+ + b\frac{\lambda}{\tau y} \geqslant 1$$

is the intersection of the regions defined by $y \leqslant y_\lambda^\star$, $\varepsilon \leqslant \varepsilon_\lambda^\star(y)$ and $\varepsilon \leqslant \varepsilon^\star(y)$ with:

$$\begin{aligned}
y_\lambda^\star &= \frac{b\lambda}{\tau} \\
\varepsilon_\lambda^\star(y) &= b\left(\frac{\lambda}{\tau} + \frac{1}{1-\tau}\right) - (y - y_\lambda^\star)\left(\frac{1}{(1-\tau)(1-\lambda)} - 1\right) \\
\varepsilon^\star(y) &= \frac{b - \tau y}{1-\tau}.
\end{aligned}$$

The low exposure regime is identified by $y_\lambda^\star$, the small shock regime by $\varepsilon_\lambda^\star(y)$ and the no-shock regime by $\varepsilon^\star(y)$, which is correctly independent from λ, encoding the network structure.

Proof. (1) If $\phi(y,\varepsilon) \geqslant 1$, i.e. $\varepsilon^\star(y)$, then the condition is fulfilled, because $\varepsilon \geqslant 0$ implies $b \geqslant \tau y$ and the l.h.s. is a convex combination of two numbers larger than 1. (2) If $b\lambda/\tau y \geqslant 1$, i.e. $y \leqslant y_\lambda^\star$ the condition is fulfilled simply because $(1-\lambda)\phi(y,\varepsilon)$ is always positive. This is also a necessary and sufficient condition when $\phi(y,\varepsilon) \leqslant 0$ (3) If

$\phi(y,\varepsilon) \in [0,1]$ then we have straightforwardly the condition $\varepsilon \leqslant \varepsilon^{\star}_{\lambda}(y)$, since

$$
\begin{aligned}
& (1-\lambda)\phi(y,\varepsilon) + b\frac{\lambda}{\tau y} \geqslant 1 \implies (1-\lambda)\left((1-\tau)(1-\frac{\varepsilon}{y}) + \frac{b}{y}\right) + b\frac{\lambda}{\tau y} \geqslant 1 \\
\implies \quad & (1-\lambda)\left((1-\tau)(y-\varepsilon)+b\right) + \frac{b\lambda}{\tau} \geqslant y \\
\implies \quad & y(1-(1-\lambda)(1-\tau)) + \varepsilon(1-\lambda)(1-\tau) \leqslant b\left((1-\lambda)+\frac{\lambda}{\tau}\right) \\
\implies \quad & (y-y^{\star}_{\lambda})(1-(1-\lambda)(1-\tau)) + \varepsilon(1-\lambda)(1-\tau) \leqslant b\left((1-\lambda)+\frac{\lambda}{\tau}(1-\lambda)(1-\tau)\right)
\end{aligned}
$$

This must be a stronger condition if instead $\phi(y,\varepsilon) \geqslant 1$ and is also a stronger condition when $\phi(y,\varepsilon) \leqslant 0$, since in this case

$$
(1-\lambda)\min(\phi(y,\varepsilon),1)^{+} + b\frac{\lambda}{\tau y} \geqslant (1-\lambda)\phi(y,\varepsilon) + b\frac{\lambda}{\tau y} \geqslant 1.
$$

For this reason, the desired region is just the intersection of (1), (2) and (3), together with the conditions of positive ε and y. □

3.1 Ring Network

We follow the same analysis of the previous section (i.e. in absence of CoCo). Again, $y_{ij} = y(\delta_{j,i+1} + \delta_{j,i-1})$ and $z = (a-\varepsilon, a, \ldots, a)$. We assume no triggers in absence of shocks, i.e.:

$$
\frac{E_i}{V_i} = \frac{a-s}{a+y} \geqslant \tau \implies (1-\tau)a - s \geqslant \tau y \geqslant 0. \tag{11}
$$

Moreover, we consider the situation where the size of the shock is at least responsible of the first bank triggering, i.e.

$$
\frac{a-\varepsilon-s}{a+y} \geqslant \tau \implies \varepsilon \geqslant \varepsilon^{\star}(y,\tau) = (a+y) - \frac{s+y}{1-\tau}, \tag{12}
$$

where $\varepsilon^{\star}(y,\tau$ coincides with the no-shock threshold of Proposition 3.2. Since $\phi^{\star}_i = 1 \implies \phi^{\star}_{i+1} = 1$, see Proposition 3.1, we can define as in the previous case:

$$
\bar{n}(\phi^{\star}) = \max\{i : \phi^{\star}_i < 1\} \tag{13}
$$

as the number of non-triggered banks. As soon as $\bar{n} < n$, it should be $\phi_n = 1$ and thus:

$$
\begin{aligned}
\phi_1 &= f_{y,s}((1-\tau)(y+a-\varepsilon)) \\
\phi_i &= (1-\tau)\phi_{i-1} + \frac{(1-\tau)a-s}{y} = (1-\tau)^{i-1}\phi_1 + \frac{(1-\tau)a-s}{y}\sum_{k=0}^{i-2}(1-\tau)^k \quad i = 2,\ldots,\bar{n} \\
\phi_i &= 1, \quad i > \bar{n}.
\end{aligned} \tag{14}
$$

Defining $\lambda_\tau^r = 1-(1-\tau)^{n-1}$, this solution holds as soon as:

$$\phi_n = (1-\lambda_\tau^r)\phi_1 + \lambda_\tau^r \frac{(1-\tau)a-s}{\tau y} \geqslant 1. \tag{15}$$

The previous condition is satisfied if $y \leqslant y_r^\star(\tau)$ or $\varepsilon \leqslant \varepsilon_r^\star(y,\tau)$, where (Proposition 3.2, with $b=(1-\tau)a-s$ and $\lambda = \lambda_\tau^r$),

$$\begin{aligned} y_r^\star(\tau) &= \frac{\lambda_\tau^r}{\tau}\left[(1-\tau)a-s\right] & (16)\\ \varepsilon_r^\star(y,\tau) &= \left(\frac{\lambda_\tau^r}{\tau}+\frac{1}{1-\tau}\right)\left[(1-\tau)a-s\right]-(y-y_r^\star)\left(\frac{1}{(1-\tau)(1-\lambda_\tau^r)}-1\right) & (17)\end{aligned}$$

In the limit $\tau \to 0$, since $\frac{\lambda_\tau^r}{\tau} \to (n-1)$, we retrieve the results of the previous section $y_r^\star(\tau) \to y^\star = (n-1)(a-s)$ and $\varepsilon_r^\star(y,\tau) \to \varepsilon^\star = n(a-s)$. We can compute the extension of the triggering contagion as $\phi_{\bar{n}} = 1$, bringing to:

$$E(\phi^\star) = \bar{n}(\phi^\star)/n = \frac{\log\left((\phi_\infty - 1)/(\phi_\infty - \phi_1)\right)}{n\log(1-\tau)}, \tag{18}$$

where $\phi_\infty = \frac{(1-\tau)a-s}{\tau y}$ and ϕ_1 as in (14).

3.2 Complete network

In this case, we can again make the ansatz

$$\phi_i^\star = \begin{cases} \phi_s^\star & \text{if } i=1, (\text{ shocked bank}) \\ \phi_{ns}^\star & \text{if } i>1, (\text{ non shocked bank}), \end{cases} \tag{19}$$

where $(\phi_s^\star, \phi_{ns}^\star)$ the solution of:

$$\begin{aligned} \phi_s &= f_{ys}((1-\tau)(a-\varepsilon+y\phi_{ns})) \\ \phi_{ns} &= f_{ys}((1-\tau)(a+\frac{y}{n-1}\phi_s + y\frac{n-2}{n-1}\phi_{ns}). \end{aligned}$$

In the safe case, where the trigger doesn't propagate through the entire network, the solution must be

$$\begin{aligned} \phi_s^\star &= f_{ys}((1-\tau)(a-\varepsilon+y)) \quad \phi_{ns}^\star = \min(1,\phi_{ns})^+ = 1 \\ \phi_{ns} &= \frac{(1-\tau)a-s}{y} + \frac{1-\tau}{n-1}\phi_s + \frac{(1-\tau)(n-2)}{n-1}\phi_{ns} \\ &= (1-\lambda_\tau^c)\phi_s + \frac{\lambda_\tau^c}{\tau}\left[(1-\tau)a-s\right], & (20)\end{aligned}$$

where we have defined

$$\lambda_\tau^c = \frac{\tau(n-1)}{1+\tau(n-2)}. \tag{21}$$

This solution exists if $\phi_{ns} \geqslant 1$, i.e. again using Proposition 3.2 with $b = (1-\tau)a - s$ and $\lambda = \lambda_\tau^c$, if and only if $y \leqslant y_c^\star(\tau)$ or $\varepsilon \leqslant \varepsilon_c^\star(y,\tau)$, where

$$y_c^\star(\tau) = \frac{\lambda_\tau^c}{\tau}[(1-\tau)a - s] \tag{22}$$

$$\varepsilon_c^\star(y,\tau) = \left(\frac{\lambda_\tau^c}{\tau} + \frac{1}{1-\tau}\right)[(1-\tau)a-s] - (y - y_c^\star)\left(\frac{1}{(1-\tau)(1-\lambda_\tau^f)} - 1\right). \tag{23}$$

We have

$$\lim_{\tau\to 0} y_c^\star(\tau) = \lim_{\tau\to 0} y_r^\star(\tau) = y^\star = (n-1)(a-s) \tag{24}$$

and

$$\lim_{\tau\to 0} \varepsilon_c^\star(y,\tau) = \lim_{\tau\to 0} \varepsilon_r^\star(y,\tau) = \varepsilon^\star = n(a-s). \tag{25}$$

4 Cocos with equity liquidation

4.1 Ring Network

Computations similar to those in the previous section bring to

$$\varepsilon_r^\star(\tau,\eta) = [(1-\tau)(a+y) - (s+y)]\left(\frac{(1-\eta)(1-C(\eta,\tau)^n)}{C^n(\eta,\tau)(1-C(\eta,\tau))}\right) \tag{26}$$

with $C(\eta,\tau) = (1-\eta)(1-\tau)$. In fact as soon as $\phi_n = 1$ we have $\forall i \leqslant \bar{n}$

$$\begin{aligned}\phi_i &= \eta + (1-\eta) f_{y,s}((1-\tau)h(\phi_{i-1})) = A + C\phi_{i-1} \\ &= C^{i-1}\phi_1 + A\sum_{k=0}^{i-2} C^k = C^{i-1}\phi_1 + A\frac{1-C^{i-1}}{1-C}\end{aligned}$$

with $A = \eta + (1-\eta)b/y$ and $C = C(\eta,\tau) = (1-\eta)(1-\tau)$. The critical threshold in the ring network is the solution of the equation $\phi_n(\varepsilon) = 1$, i.e.

$$C^{n-1}\phi_1(\varepsilon) + A\frac{1-C^{n-1}}{1-C} = 1 \tag{27}$$

where $\phi_1(\varepsilon) = A + C(1-\varepsilon/y)$. Straightforward manipulations bring equation (26)

4.2 Complete Network

In the case of the complete network we have that

$$\varepsilon_c^\star(\tau,\eta) = [(1-\tau)(a+y)-(s+y)]\left(\frac{n-\tau-\eta(1-\tau)}{(1-\eta)(1-\tau)^2}\right). \tag{28}$$

In fact in this case the critical shock is the solution of the equation $\phi_{ns}(\varepsilon) = 1$, i.e.

$$\phi_{ns}(\varepsilon) = \eta + (1-\eta) f_{ys}((1-\tau)(a+\frac{y}{n-1}\phi_s(\varepsilon)+y\frac{n-2}{n-1})) = 1, \tag{29}$$

where

$$\phi_s(\varepsilon) = \eta + (1-\eta)\frac{(1-\tau)(a-\varepsilon+y)-s}{y}. \tag{30}$$

Solving in ε equation (28) follows straightforwardly.

5 Networks with macroscopic structures

In this Section we analyze the behavior of the contagion in the case of more realistic interbank networks, in particular with some level of degree heterogeneity induced by a macroscopic structure. To this aim we have generated from the well known Stochastic Block Model[3] random networks with a given community or core-periphery structure. For simplicity, banks are divided in two groups, and links are introduced with a probability that depends on the groups the two nodes belong. For the community structure case, nodes of the same group have a probability p_{in} to be connected, while there's a probability p_{out} to connect two nodes from different groups.

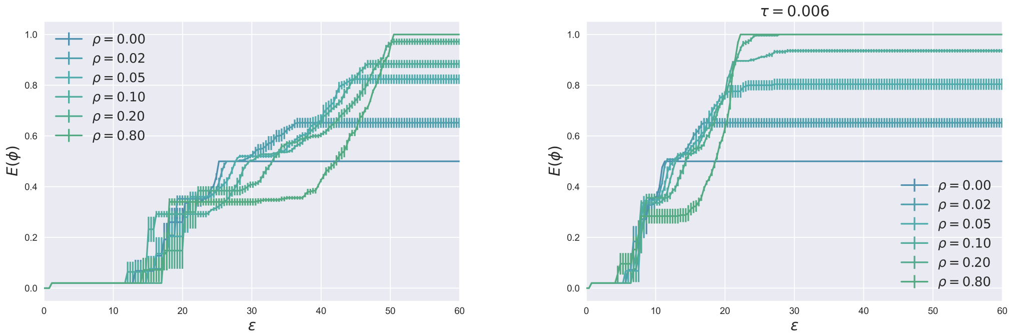

Fig S1: Extent of contagion in a network with two assortative communities of banks.

In Fig. S1 the extent of contagion (in presence and absence of Cocos) is shown. In this experiment we fix the mean degree of the network, and we change the strength of the community structure with a parameter

$\rho \in [0,1]$ such that $p_{out} = \rho p_{in}$. For $\rho \sim 1$, the structure is weak, and we retrieve the result of the paper for the corresponding level of connectivity. When $\rho = 0$, the communities are disconnected, and we retrieve the result of the paper but for a halved system. For $0 < \rho < 1$, it is evident a first jump of the contagion to the first neighbors of the shocked bank, followed by a slower propagation of the distress that slowly invade also the second communities. Note that, both the speed of the contagion and its maximum extent, depend on ρ and, therefore, on the two levels of connectivity p_{out} and p_{in}: a smaller p_{out} can reduce the maximum impact of the contagion but, at the same time, can increase the sensibility to the shock size.

The situation is pretty similar in the case of networks with a core-periphery structure. In this case the two groups of banks represent the core (big banks usually too big to fail banks) and the periphery (small banks). Core banks are connected among them with probability p_c. A peripheral bank can be connected to a core bank with probability p_{pc}, and two peripheral banks are connected with probability p_p, with

$$p_c \geqslant p_{pc} \geqslant p_p.$$

In our experiment, shown in Fig. S2, we fix again as before the mean connectivity of the network and then we assumed $p_p = 0$, $p_{pc} = \rho p_c$, $\rho \in [0,1]$.

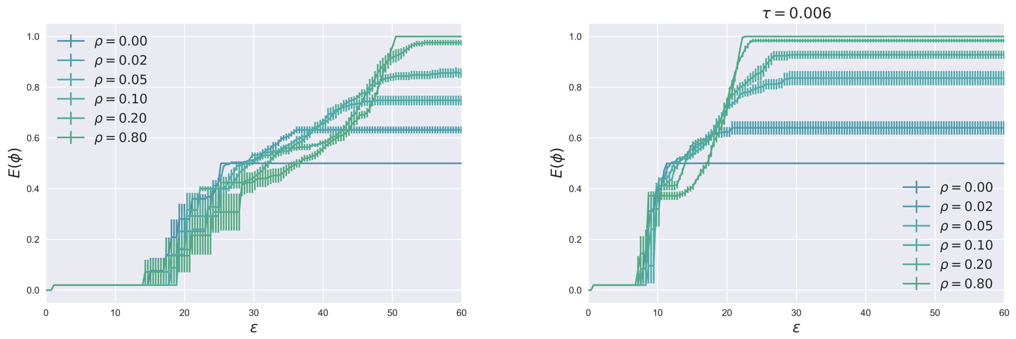

Fig. S2: Extent of contagion in a network of banks with a core-periphery structure, where a bank in the core is shocked.

The Figure shows the extent of contagion and Cocos triggering in the case where a core bank experienced a liquidity shock. The distress propagation is very similar to that of a networks with two assortative communities. Obviously, the situation is completely different when a peripheral bank suffers a liquidity distress. In this situation, the trigger of a contagion is typically very difficult if p_{pc} is too small.

From this analysis it appears that the results explained in the main manuscript are highly robust. First order effects on the extent of contagion can be explained in terms of the (few) different levels of connectivities in the

networks.

References

1. Acemoglu, D., Ozdaglar, A. & Tahbaz-Salehi, A. Systemic risk and stability in financial networks. *Am. Econ. Rev.* **105**, 564–608 (2015).

2. Eisenberg, L. & Noe, T. H. Systemic risk in financial systems. *Manag. Sci.* **47**, 236–249 (2001).

3. Decelle, A., Krzakala, F., Moore, C. & Zdeborová, L. Asymptotic analysis of the stochastic block model for modular networks and its algorithmic applications. *Phys. review E* **84**, 066106 (2011).
